# Suspected Spontaneous Aqueous Humor Misdirection Syndrome in a Boston Terrier

**DOI:** 10.1155/2020/1092562

**Published:** 2020-05-19

**Authors:** Ashley E. Zibura, Michael G. Davidson, Hans D. Westermeyer

**Affiliations:** Comparative Ophthalmology, Department of Clinical Sciences, College of Veterinary Medicine, North Carolina State University, 1060 William Moore Drive, Raleigh, NC 27607, USA

## Abstract

An eight-year-old female spayed Boston Terrier presented to the North Carolina Veterinary Hospital with glaucoma in the left eye (OS). Initial ophthalmic examination revealed moderate ocular hypertension, a diffusely and markedly shallow anterior chamber with anteriorly displaced iris and lens, vitreal prolapse, and a normal iridocorneal angle (ICA) morphology. The patient displayed a paradoxical response to topical latanoprost with an increase in intraocular pressure. These examination findings led to a putative diagnosis of spontaneous aqueous humor misdirection syndrome (AHMS). The patient was successfully managed with topical carbonic anhydrase inhibitors (CAIs) and apraclonidine for eight months until progressive ulcerative keratitis necessitated enucleation of the affected globe. Histopathology and high-field magnetic resonance imaging (MRI) of the enucleated globe did not identify an underlying cause for the glaucoma. This case suggests that AHMS should be considered in dogs presenting with a shallow anterior chamber, vitreal prolapse, increased intraocular pressure, and no other causes of glaucoma.

## 1. Introduction

Aqueous humor misdirection syndrome (AHMS) is a rare form of secondary glaucoma thought to result from posteriorly misdirected aqueous humor which becomes trapped in the vitreous chamber [1, 2]. This leads to the anterior displacement of the iris and lens, resulting in progressive pupillary block and increased intraocular pressure (IOP) [1, 2].

Also known as malignant glaucoma, AHMS is relatively rare. Spontaneous AHMS has been described primarily in geriatric cats. These cats typically present with a uniformly shallow anterior chamber, anterior displacement of vitreous, and modest increases in IOP [1, 2]. Initially, the iridocorneal angle (ICA) is open [2], but with progressive anterior displacement of the iris-lens diaphragm, angle narrowing and ciliary cleft collapse can develop [1, 2].

Induced aqueous humor misdirection syndrome (AHMS) has been reported in a dog [3] and a llama [4] following phacoemulsification, and in humans following penetrating keratoplasty [5], phacoemulsification [6], and neodymium: YAG laser posterior capsulotomy [7]. However, spontaneous AHMS has not yet been described in dogs.

This report describes a suspected case of spontaneous AHMS in a canine patient.

## 2. Case Presentation

### 2.1. Signalment and History

An eight-year-old female spayed Boston Terrier was presented for the evaluation of acute glaucoma OS. An IOP of 38 mmHg OS (13 mmHg in the right eye (OD)) was noted 24 hours earlier by the referring veterinarian who prescribed three times daily topical dorzolamide OS (dorzolamide hydrochloride 2% ophthalmic solution, Sandoz Inc., Princeton, NJ, USA). Three years prior, a conjunctival pedicle graft had been placed to treat a deep stromal corneal ulcer OS. The owners reported no other past health problems.

### 2.2. Initial Ophthalmic Examination

**Figure 1:**
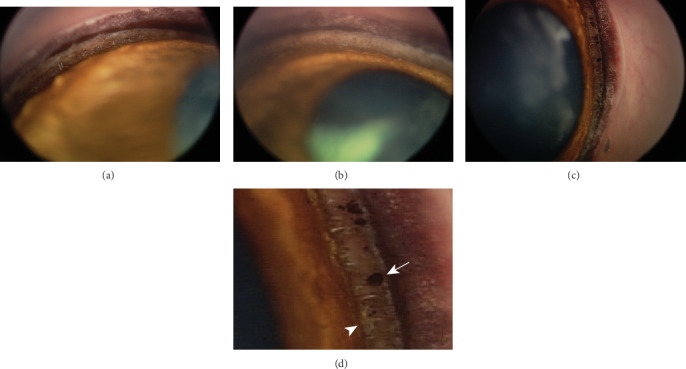
Gonioscopy images of both eyes with an ICA Grade 2-3 and sparse areas of fibrae latae. (a) View of one quadrant OS. (b) View of one quadrant OD. (c) View of a different quadrant OD. (d) Magnified view of a sector of the ICA OD showing foci of pigment (white arrow) within the trabecular meshwork and small foci of fibrae latae (white arrowhead) at the opening of the ICA.

On presentation, neuroophthalmic examination was unremarkable with intact palpebral reflex, menace response, direct and indirect pupillary light reflexes (PLR), and dazzle reflex. On slit lamp biomicroscopy (Kowa SL-15, Tokyo, Japan) of the left eye, very mild faint corneal fibrosis was noted as the remnant of the patient's previous conjunctival pedicle graft. The anterior chamber was uniformly, markedly shallow such that the iris nearly contacted the corneal endothelium. The pupil was slightly larger than midrange. No iridodonesis or phacodonesis was noted. Vitreal strands extended through the pupil into the shallow anterior chamber, filling the ventral 1/3 of the pupil opening. An early immature anterior cortical cataract was also noted, affecting roughly 20% of the lens. IOP (Tono-Vet, iCare, Vantaa, Finland) measured 32 mmHg OS, compared to 8 mmHg OD. Binocular indirect ophthalmoscopy (Vantage LED Plus, Keeler, Malvern, PA, USA, and 20D or 28D condensing lenses, Volk Optical Inc., Mentor, OH, USA) OD was unremarkable and was not performed OS. The gonioscopic evaluation (RetCam, Clarity Medical Systems, Pleasanton, CA, USA) of the right eye revealed a normal iridocorneal angle opening, ranging from grade 2 to 3 throughout the eye's circumference [8], and minimal pectinate ligament dysplasia characterized by a few small sectors of fibrae latae (Figure 1). Direct gonioscopy of the left eye was not performed at this initial examination due to the markedly shallow anterior chamber.

### 2.3. Initial Response to Therapy

The left eye was treated with several doses of alternating dorzolamide hydrochloride 2%/timolol maleate 0.5% (Cosopt, Bausch & Lomb, Rochester, NY, USA) and latanoprost 0.005% ophthalmic solution (Bausch & Lomb). After 30 minutes of therapy, the IOP OS increased to 42 mmHg and moderate miosis was noted. A drop of tropicamide 1% (Bausch & Lomb) was administered OS in an attempt to relieve the suspected pupillary block component of the patient's ocular hypertension. Thirty minutes later, the IOP OS was 40 mmHg with no significant changes in pupil size noted. Four additional doses of Cosopt and three additional doses of latanoprost were administered over the next 90 minutes in an attempt at achieving ocular hypotension; however, this resulted in an IOP of 48 mmHg. At that time, two doses of mannitol (20%, 1 gram/kg slow IV push through an in-line filter over 30 min, Hospira Inc., Lake Forest, IL, USA) were administered 90 minutes apart. Four hours later, the IOP OS had decreased to 10 mmHg. Cosopt (q8h OS) was continued overnight. The following morning, the IOP OS was 8 mmHg with a notably deeper anterior chamber depth. The dog was discharged with Cosopt (q8h OS) and tramadol (2.5 mg/kg PO q8-12h PRN for 5 days, Amneal Pharmaceuticals, Bridgewater, NJ, USA).

### 2.4. Subsequent Ophthalmic Examination and Treatment

Three days later, the IOP OS was 17 mmHg OS (10 mmHg OD) with normal anterior chamber depth, and similar findings to those noted at discharge. Indirect ophthalmoscopy revealed a normal fundus and gonioscopy of the left eye paralleled that of the right eye with a normal iridocorneal angle opening ranging from grade 2 to 3 throughout the eye's circumference and minimal pectinate ligament dysplasia characterized by a few small sectors of fibrae latae (Figure 1). Sixteen days later, the IOP OS was 19 mmHg (11 mmHg OD) with a slightly shallow anterior chamber and vitreous strands filling the 50% of the pupil opening. Cosopt (q8h OS) was continued, and apraclonidine (0.5% ophthalmic solution, 2 drops 2 minutes apart twice daily OS, Akorn Inc., Lake Forest, IL, USA) was added for adjunctive therapy. Two weeks and 5 months later, the IOP OS was 10 mmHg and 13 mmHg, respectively (10 mmHg and 14 mmHg OD, respectively).

Eight months after initial evaluation, the dog's left eye became acutely red, cloudy, and painful. Menace response, direct pupillary light reflex (PLR), and indirect PLR (from OD to OS) were absent OS. Indirect PLR (from OS to OD) and dazzle reflex OS were intact. Biomicroscopic examination OS revealed moderate conjunctival hyperemia with scleral injection, superficial corneal neovascularization, multifocal areas of corneal edema with corneal bullae, and multifocal punctate areas of superficial corneal ulceration. The anterior chamber was uniformly shallow, mirroring the chamber depth noted at the time of initial glaucoma diagnosis. The iris leaflets were uniformly anteriorly displaced. Anterior uveitis with a concurrent hypermature cataract was noted with a miotic, nonresponsive pupil and 2+ aqueous flare. An incipient cortical cataract was noted OD with no other changes. The IOP OS was 34 mmHg (10 mmHg OD). The patient was treated with 1 drop of Cosopt ophthalmic solution OS every 5 minutes for a total of 30 minutes. Subsequent IOP reading OS was 24 mmHg. The patient was discharged on continued Cosopt therapy three times daily OS, apraclonidine ophthalmic therapy increased to three times daily OS, hypertonic saline 5% ophthalmic solution (Muro-128, Bausch & Lomb) four times daily OS, flurbiprofen sodium 0.03% ophthalmic solution (Bausch & Lomb) four times daily OS, ofloxacin 0.3% ophthalmic solution (Rising Pharmaceuticals Inc., Saddle Brook, NJ, USA) every 2 hours during waking hours, and oral carprofen (2.2 mg/kg, Rimadyl, Zoetis, Kalamazoo, MI, USA) by mouth twice daily. Three days later, the patient's neuroophthalmic examination remained static with an IOP of 19 mmHg OS (13 mmHg OD), and a 3-4 mm diameter midstromal corneal ulcer with 50% stromal loss. An aerobic culture of the ulceration identified a coagulase-negative staphylococcus species and Corynebacterium species. Five days later, the ulcer had progressed to 70% stromal loss and the owners elected to enucleate OS. At the time of globe removal, performed via a subconjunctival approach, normal retrobulbar and extraocular anatomy was noted. In an effort to try to further investigate the ocular pathology, the excised globe was submitted for high-field magnetic resonance imaging and histopathologic analysis.

### 2.5. MRI Methods

The enucleated left globe was fixed in 10% formalin (Azer Scientific, Morgantown, PA, USA) for twenty days. The eye was then carefully transferred to a formalin-filled 60 milliliter syringe (Becton Dickinson, Franklin Lakes, NJ, USA). Imaging was performed using a Bruker BioSpec 94/30USR 9.4 T horizontal bore scanner MRI (Bruker Corporation, Billerica, MA, USA). The eye was imaged in a transverse plane overnight, and images were acquired with the ParaVision software (Bruker Corporation).

### 2.6. MRI Findings

**Figure 2:**
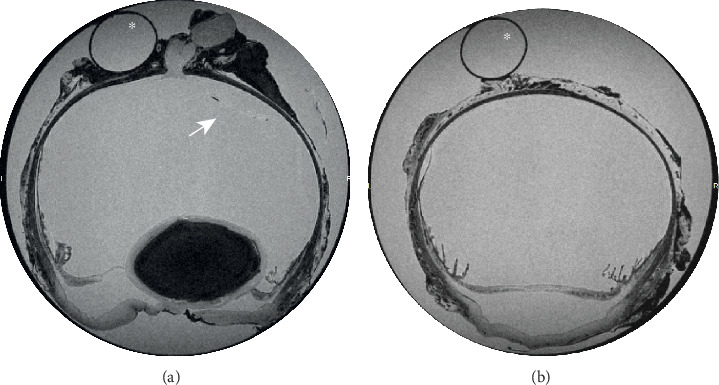
(a) Axial and (b) peripheral transverse sections of the enucleated canine globe (OS) imaged with high field 9.4 T MRI. Note the corneal stromal loss consistent with the patient's bacterial keratitis, the shallow anterior chamber with notable loss of aqueous humor, and the multifocal undulation of the neurosensory retina (white arrow) consistent with multifocal retinal detachment which occurred artifactually, subsequent to globe fixation and processing. The white asterisks denote the plastic drinking straw used for globe positioning during image acquisition.

Focal disruption in axial corneal epithelium was noted with moderate focal stromal loss. The corneal curvature had a diffusely undulating appearance. The anterior chamber was markedly shallow secondary to dehydration in formalin. However, the ciliary cleft appeared open. The lens appeared slightly posteriorly subluxated. The vitreous chamber appeared intact with multifocal undulation of the neurosensory retina consistent with multifocal retinal detachment. The optic nerve head appeared intact and unremarkable (Figure 2).

### 2.7. Histopathology Findings

**Figure 3:**
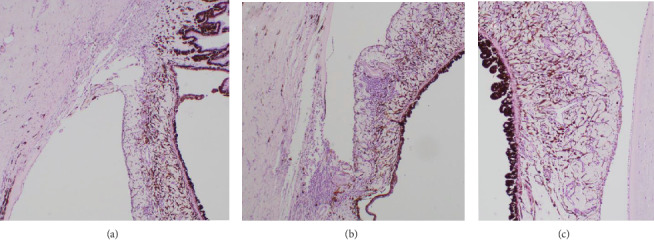
Histopathology images of the enucleated canine globe (OS) with hematoxylin and eosin (H&E) stain. (a) Image of the nondependent ICA demonstrating an open angle and lack of goniodysgenesis at 4x magnification, thus ruling out primary glaucoma as the cause of this patient's glaucoma. (b) Image of the dependent ICA demonstrating an open angle, lack of goniodysgeneis, moderate lymphoplasmacytic infiltrate in the iris, ciliary body, and trabecular meshwork, and mild preiridal fibrovascular membrane (PIFM) formation, viewed at 4x magnification. This very mild degree of inflammation is incongruent with an 8 month history of uveitis-driven glaucoma, thus supporting a differential cause for this patient's glaucoma, such as AHMS. (c) Image of the iris leaflet with scant lymphoplasmacytic infiltration and scant PIFM.

The sections of the globe were routinely stained with hematoxylin and eosin. Grossly, the lens was posteriorly luxated. There was moderate keratinization of the corneal epithelium. The peripheral superior aspect of the cornea had marked fibrosis and vascularization of the superficial corneal stroma. There was a marked loss of the axial corneal stroma associated with moderate edema and mild fibroplasia, in addition to focally extensive doubling of Descemet's membrane with the formation of an axial retrocorneal membrane. There was moderate lymphoplasmacytic infiltrate in the iris, ciliary body, and trabecular meshwork, with a mild preiridal fibrovascular membrane (PIFM). Even after multiple deeper sections of the block, the iridocorneal angles appeared normal, and no lesions consistent with goniodysgenesis were identified (Figure 3). There was moderate liquefaction of the cortical lens fibers with the formation of morgagnian globules, multifocal areas of mineralization, and posterior migration of the lens epithelium. Alcian blue stain revealed the moderate degeneration of the vitreous with accumulation of dense aggregates of vitreal matrix adjacent to and associated with the equatorial lens capsule. The number of ganglion cells was mildly decreased, and the optic nerve presented moderate gliosis. The retinal and optic nerve lesions were very mild, and thus definitive confirmation of the presence of glaucoma was not possible.

### 2.8. Follow-up

At recheck examination 7 days following enucleation, the patient's surgical incision was intact and healing well, and her right eye remained comfortable and visual. On follow-up communication with her owners 10 months following surgery, her right eye remained comfortable. Her cataract OD had progressed, causing moderate visual impairment; however, no clinical signs of glaucoma were observed.

## 3. Discussion

The patient's clinical presentation and ophthalmic examination findings mirror those reported in cases of feline spontaneous AHMS [1, 2, 9]. Specifically, a distinctly shallow anterior chamber, anterior displacement of vitreous, a normal iridocorneal angle, and a modest increase in IOP. Additionally, iridocorneal anomalies associated with primary angle closure glaucoma (PACG) were ruled out gonioscopically and histopathologically, and no other findings associated with secondary glaucoma were identified. Moreover, the rise in IOP in response to latanoprost implicates pupillary block as at least one of the likely mechanisms generating increased IOP in this patient, possibly as a component of ciliovitreolenticular block as previously described in cases of feline AHMS [2]. The dog of this report also manifested signs of vitreous humor degeneration with anterior chamber presentation of vitreous. This is thought to contribute to the pathogenesis of AHMS in humans [10]. A marked increase in IOP, from 32 to 42 mmHg, after latanoprost administration was noted in the present case. This 31% IOP increase is significantly outside the range of potential variation from tonometry [11–13] and is likely due to exacerbation of pupillary block from the shallow anterior chamber and iris-lens-vitreous anterior displacement. The IOP decreased, albeit very slightly (from 42 to 40 mmHg), following the administration of tropicamide which was given with the aim of releasing the patient's suspected pupillary block. Tropicamide was unsuccessful at relieving the patient's ocular hypertension and the pupil remained miotic. Thus, in this patient, the effects of tropicamide were likely overridden by the miotic effects of latanoprost. It should also be noted that subsequent doses of latanoprost resulted in progressive IOP elevation, reaching 48 mmHg prior to mannitol treatment. This provides further evidence to support the occurrence of pupillary block in this patient as a likely primary mechanism driving glaucoma.

Other mechanisms by which the flow of aqueous humor can be blocked at the level of the ciliary body, anterior vitreous face, and/or pupil include: iris bombe, iridociliary cysts, intraocular neoplasia, lens sub/luxation, intumescent lens, and spherophakia [1]; however, all of these disease processes were definitively ruled out in this case through ophthalmic examination, histopathologic globe analysis, and high-field MRI of the enucleated globe. A block of aqueous flow distal to the level of the ICA is highly unlikely in this case based on the absence of overt uveitis at initial presentation, histopathologically normal trabecular meshwork and ciliary cleft, and normal retrobulbar anatomy noted at the time of enucleation. While gonioscopy cannot rule out the possibility of primary open angle glaucoma (POAG), this form of glaucoma has neither been reported in the Boston Terrier breed nor has it been reported in association with anterior iris displacement and vitreal prolapse [14]. Moreover, an elevated intraocular pressure was not recorded in the opposite eye as of the last follow up—approximately 1.5 years after initial pressure elevation in the first eye.

It seems unlikely uveitis was the cause of the initial pressure elevations, even though clinical uveitis was observed prior to enucleation and histologic evidence of uveitis was noted. There were no clinical signs of uveitis noted at the time of glaucoma diagnosis, or at any point over the following eight months. Additionally, if uveitis were the driving factor for glaucoma for eight months, more robust uveal inflammation and PIFM would be expected. Thus, the very mild inflammatory infiltrate of the dependent iridocorneal angle and the thin PIFM noted on histopathologic examination is incongruent with an 8-month history of uveitis-driven glaucoma. Therefore, AHMS is a more plausible differential for the initial glaucoma. It is much more likely the uveitis noted prior to enucleation was unrelated to the initial glaucoma and was a separate process driven by a combination of ulcerative keratitis and a hypermature cataract.

Treatment with dorzolamide/timolol and apraclonidine adequately controlled the patient's IOP for eight months in the case presented here. However, the patient's uveitis and concurrent ocular changes towards the end of the disease course were likely driving glaucoma through different secondary mechanisms. Outside of the patient's complicating end-stage factors, it is unclear what the true duration of response to AHMS therapy the patient would have experienced. Conservative management with topical carbonic anhydrase inhibitor medications is the mainstay of therapy for feline spontaneous AHMS [1, 2], with phacoemulsification, anterior vitrectomy, posterior capsulotomy, and/or endocyclophotocoagulation considered in refractory cases [1, 2, 9]. In feline patients, AHMS tends to progress slowly, although most cases ultimately become refractory to treatment with a guarded to poor overall prognosis for vision or IOP control [1, 2].

While systematic clinical exclusion of other differentials validates a diagnosis of spontaneous AHMS in the presented case, definitive confirmation was not possible. AHMS is typically confirmed through observation of pools of aqueous humor within the vitreous chamber or between the vitreous and the retina [1, 2]. However, the majority of the aqueous fluid in the globe diffused out during processing with formalin as a result of its hyperosmolar properties [15], thereby preventing observation of pools of aqueous fluid in the posterior segment on histopathologic or MRI analysis. This loss of fluid also appears to have contributed to diffuse volume contraction of the globe, leading to artefactual lens subluxation and multifocal retinal detachments noted during ex vivo analysis. The vitreal condensation initially described in cats with AHMS [2, 16] was not seen histologically in the current report either. However, the last episode of elevated intraocular pressure due to suspected AHMS occurred more than 8 months prior to enucleation. It is possible that these bands were present during the episodes of elevated intraocular pressure but resolved over time.

Additional diagnostic tests which should be considered for thorough evaluation of cases of suspected AHMS include streak retinoscopy to evaluate for potential refractive errors subsequent to anterior displacement of the iris-lens diaphragm and vitreous body [6, 17, 18], high-resolution ocular ultrasonography (HRUS) or ultrasound biomicroscopy (UBM) to better quantify the degree of anterior chamber narrowing and evaluate for posterior segment foci of aqueous humor fluid [6, 18, 19], and optical coherence tomography (OCT) to further quantify anterior and posterior segment abnormalities [19, 20]. These tests were not performed in the presented case; however, they may have provided insight to further support a diagnosis of spontaneous AHMS and should be considered for workup of future cases. Clinical B-scan ocular ultrasonography with a 10 or 12 MHz probe was not performed due to the assessed low clinical yield of identifying pools of aqueous fluid in the vitreal cavity with this probe frequency. HRUS or UBM provides higher resolution which is valuable for diagnosing more subtle lesions [21]. As a result, HRUS and UBM are the preferred ultrasonic imaging modalities for AHMS diagnosis in humans [6, 17–19] and have been used successfully in the diagnosis of feline AHMS [2].

It is important to note that although detecting pockets of aqueous humor is considered a positive diagnostic finding in AHMS, the lack of this finding on HRUS or the absence of this test does not preclude diagnosis. In fact, only 9 of the 32 cats in the seminal publication describing spontaneous feline AHMS [2] underwent HRUS. Additionally, the authors of that study suggest aqueous humor may become diffusely distributed throughout the vitreous. Having HRUS showing pockets of aqueous humor within the vitreous would have helped confirm the etiology of pressure elevations in the presented case. However, based on the present literature available on the subject [4–7, 17, 18, 20, 22–24], the negative predictive value of HRUS in diagnosing AHMS is low. In other words, an ultrasound had been performed and no aqueous humor pockets were observed, AHMS could not be ruled out. Thus, the lack of ultrasound examination in this case report, although a weakness, does not represent a failure to positively diagnose this syndrome in a dog.

This report describes a presumptive case of spontaneous AHMS in a dog. While not previously reported in dogs, this syndrome should be considered as a differential diagnosis in cases of glaucoma with atypical clinical findings, open ICA morphology, and paradoxical response to standard medical therapy.
